# Intraductal papillary mucinous neoplasm of the accessory pancreatic duct in the pancreas uncinate process: A case report

**DOI:** 10.1097/MD.0000000000033840

**Published:** 2023-05-26

**Authors:** Tao Guo, Ya Liu, Zhu Yang, Jing Li, Kun You, Dejun Zhao, Sujuan Chen, Cong Li, Pei Yang, Hongqiang Hu, Hao Zhang

**Affiliations:** a Department of Hepatopancreatobiliary Surgery, Foguang Hospital of Emei Mountain, Emei Mountain, Si Chuan, China; b Department of Pancreatic Surgery, West China Hospital, Sichuan University, Chengdu, Si Chuan, PR China; c Department of Operating Room, West China Hospital, Sichuan University/West China School of Nursing, Sichuan University, Chengdu, Si Chuan, PR China; d Department of Oncology, Foguang Hospital of Emei Mountain, Emei Mountain, Si Chuan, China.

**Keywords:** accessory pancreatic duct (APD), case report, duodenum-preserving partial pancreatic head resection (DPPHR-P), intraductal papillary mucinous neoplasms (IPMN), pancreas neoplasm, postoperative pancreatic fistula (POPF)

## Abstract

Intraductal papillary mucinous neoplasms (IPMN) of the accessory pancreatic duct (APD) are very rare and their clinical significance is not known. Here, we describe a case of IPMN originating in a branch of the duct of APD within the uncinate process of the pancreas, which initially presented with acute pancreatitis.

**Patient concerns:**

A 70-year-old man visited our medical center presenting with acute pancreatitis around the head and uncinate process of the pancreas.

**Diagnoses:**

Computer tomography scans revealed the presence of a 35-mm cystic mass-like lesion within the pancreas uncinate process communicating with a branch of the APD. The patient was diagnosed with APD-IPMN in the pancreas uncinate process accompanied by acute pancreatitis.

**Interventions:**

Conservative management of the acute pancreatitis relieved his symptoms, while duodenum-preserving partial pancreatic head resection (DPPHR-P) was performed to treat the APD-IPMN. Intraoperative exploration showed the presence of severe adhesions within the uncinate process of the pancreas and that the tumor’s “peduncle” – a branch of the duct of APD – was saddling just at the front of the main pancreatic ducts. Thus, surgical removal of the tumor required special handling of the region between the main duct (MD) and APD to protect the integrity of the main pancreatic ducts. Finally, a 35*30*15 mm IPMN was successfully removed and the MD was preserved combined with ligation from the root of the APD of the pancreas. The drainage volume of the ventral tube increased by around 20-fold in 24 hours on the fourth day after surgery. The presence of high amylase levels in the drainage discharge (40713.5 U/L) led to the diagnosis of postoperative pancreatic fistula (POPF). The drainage volume remained high for 3 days.

**Outcomes:**

The patient was discharged and POPF was successfully managed through endoscopic pancreatic duct stenting.

**Lessons:**

APD-IPMN in the pancreas uncinate process has its own characteristics of localized pancreatitis, and MD-preserving DPPHR-P not only protects the exocrine and endocrine functions of the pancreas, but it also protects the physiological and anatomical integrity. The appearance of POPF after DPPHR-P may be managed by endoscopic pancreatic duct stenting.

**Key points**

A 70-year-old man was observed with intraductal papillary mucinous neoplasms (IPMN) within the pancreas uncinate process communicating with a branch of the accessory pancreatic duct (APD) following admission for acute pancreatitis. The tumor’s “peduncle” was just saddling just at the front of the main pancreatic ducts, which was an unusual manifestation of IPMN. APD-IPMN accompanied by acute pancreatitis was diagnosed. Duodenum-preserving partial pancreatic head resection was performed to treat the APD-IPMN and to preserve the main duct. Although postoperative pancreatic fistula occurred on the fourth day after duodenum-preserving partial pancreatic head resection surgery, it can be managed by endoscopic pancreatic duct stenting successfully.

## 1. Introduction

Intraductal papillary mucinous neoplasms (IPMN) are specific pancreas cystic lesions. Since it was first reported as a distinguishable pancreatic mucus-producing neoplasm in 1982, IPMN is one of the most attractive pancreatic neoplasms in the last few decades. The clinical symptoms of IPMN vary, with a majority of the patients being asymptomatic or only having mild symptoms, like abdominal pain, fever, wasting, and diabetes. However, these symptoms are not specific to IPMN. Although the prevalence of IPMN is low, ranging from 3 to 6% in the general population, the development of modern imaging and the increase in health awareness has increased its detection.^[1,2]^ IPMN is characterized by duct dilation and excessive mucin secretion in the main duct (MD) and/or the branch duct (BD) of the pancreas. Based on these characteristics, it can be divided into MD-IPMN, BD-IPMN, and mixed-type IPMN. However, in embryogenesis, the pancreas is composed of separate dorsal and ventral buds, which converge to produce the mature pancreas. The distal part of the MD and APD are derived from the dorsal pancreas, whereas the head part of the MD arises from the ventral pancreas.^[3–6]^ Therefore, APD-IPMN in the pancreas uncinate process might be a separate entity different from the common classification of MD-IPMN, BD-IPMN, and mixed-type IPMN.^[3,7]^ APD-IPMN is very rare and its clinical significance is not known. Literature regarding APD-IPMN and its branches is scarce.

IPMN is considered to be a precancerous lesion for pancreatic cancer. When IPMN patients develop cancer, it is referred to as “IPMN with an invasive carcinoma” or “intraductal papillary mucinous carcinoma” (IPMC). IPMC accounts for about 10% of resected pancreatic cancers of ductal origin.^[1,8,9]^ According to the Fukuoka consensus, high-risk stigmata that require resection, are characterized by cysts with obstructive jaundice, enhancing mural nodule ≥5 mm and MD diameter ≥10 mm. Worrisome features that require endoscopic ultrasound include cyst size ≥ 3 cm, enhancing mural nodule < 5 mm, thickened enhanced cyst walls, MD diameter 5 to 9 mm, abrupt change in the MD diameter caliber with distal pancreatic atrophy, lymphadenopathy, an elevated serum level of CA19-9, a rapid rate of cyst growth ≥ 5 mm/2 years and history of pancreatitis.^[2,10]^ The 5-year risk of pancreatic cancer for IPMN is 49.7%, which is significantly greater than for worrisome features cysts (4.1%) and non-high-risk feature/worrisome features cysts (2–3%).^[11]^ Since MD-IPMN and mixed-type IPMN are highly associated with malignancy, surgical removal is recommended. Oyama et al reported that the three-year risk of developing pancreatic malignancy after BD-IPMN diagnosis was 3.3%, with the 5-year risk increasing to 15.0%.^[12]^ The treatment for BD-IPMN in general is relatively conserved. The main basis for the selection of therapy for BD-IPMN is the classification into “Worrisome features and “high-risk stigmata.”^[2]^ The type of surgery to be performed (either pancreaticoduodenectomy, distal pancreatectomy, or total pancreatectomy) is dependent on the location and tumor size. Since BD-IPMN is more frequently located in the pancreatic head, limited pancreatic head resection (excision, enucleation, duodenum-preserving total/partial pancreatic head resection) might be an optimal choice for IPMN without malignant tendency.^[2]^ Here we report a partial pancreatic head resection of APD-IPMN within the uncinate process of the pancreas.

## 2. Case presentation

### 2.1. Chief complaints

A 70-year-old man was referred to our medical center after suddenly presenting with middle and upper abdominal pain for 4 hours.

### 2.2. History of present illness

The patient developed middle and upper abdominal pain radiating to the back, showing persistent colicky pain, without any obvious cause 4 hours before admission. He was referred to our hospital. Rest does not relieve the pain.

### 2.3. History of past illness

He had been diagnosed with hypertension in the past.

### 2.4. Personal and family history

He had a history of smoking (a pack per day for 30 years) and drinking (pure alcohol of >72 g per day for 30 years) but had quit smoking and drinking for 0.5 years. The patient had no significant past medical history and denied any family history of tumors.

### 2.5. Physical examination

Physical examination revealed tenderness in the upper abdomen without rebound tenderness and tension. Other physical examination was normal.

### 2.6. Laboratory examinations

Blood (3116.4 U/L) and urinary (6059.6 U/L) amylase levels were significantly elevated, while tumor markers (CA19-9, CEA) were within the normal ranges on admission.

### 2.7. Imaging examinations

Computer tomography (CT) scan of the abdomen showed a 35 mm cystic mass-like lesion located in the uncinate duct of the pancreas, and that the main pancreatic duct was slightly dilated with surrounding exudation.

### 2.8. Initial diagnosis and treatment

Based on these findings, he was initially diagnosed with acute pancreatitis and space-occupying lesions in the pancreatic head. He continued fasting, and pancreatitis-associated drugs were given to inhibit pancreatic enzyme activity, enhance the microcirculation, and maintain the acid-base and electrolyte balance. After conservative management of acute pancreatitis, his symptoms got better.

### 2.9. Further diagnostic workup

**Figure 1.**
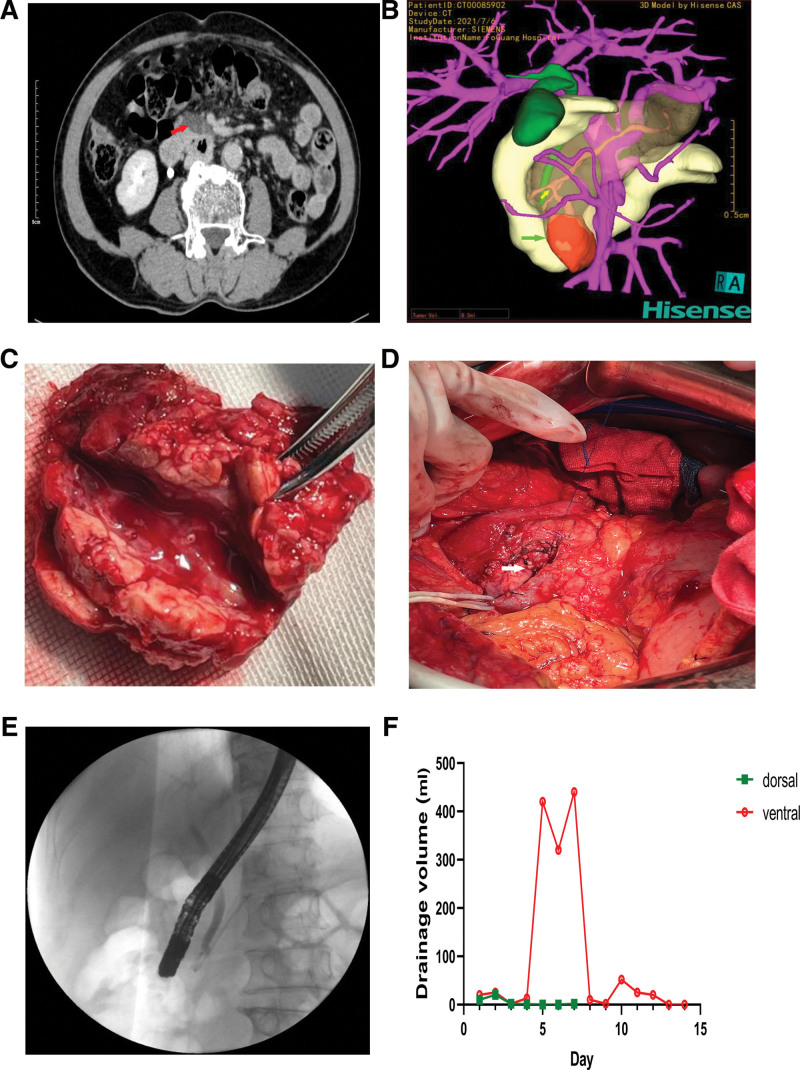
Process of intraductal papillary mucinous neoplasm of the accessory pancreatic duct diagnosis and treatment. (A and B) CT and 3D reconstructed CT shows the cystic neoplasm of the accessory pancreatic duct in the pancreas uncinate process (red arrow: a 32*29*14 mm sized solid-cystic tumor; green arrow: 3D reconstructed images of the tumor; yellow arrow: the tumor communicating with a branch of the duct of APD); (C) Postoperative specimen shows small amounts of mucin secretion; (D) The white arrow refers to the broken end of the accessory pancreatic duct; (E) X-ray image shows insertion of the pancreatic ductal stent after endoscopic retrograde cholangiopancreatography plus endoscopic sphincterotomy + endoscopic retrograde pancreatic; (F) The volume of the dorsal and ventral drainage tubes. APD = accessory pancreatic duct.

Repeat CT and 3-dimensional reconstructed CT of the pancreas revealed a 32*29*14 mm sized cystic mass within the uncinate process of the pancreas communicating with a BD of the APD (Fig.1 A and B). The MD diameter was 3 mm which was normal. The upper margin of this cystic mass was close to anterior inferior pancreaticoduodenal vein and the tumor was just in front of the boundary between the MD and the APD. These findings combined with the medical history of the patient and other examinations confirmed the presence of BD-IPMN of the APD with acute pancreatitis, which was an indication for surgery.^[2,13]^

### 2.10. Final diagnosis

The patient was diagnosed with APD-IPMN in the pancreas uncinate process accompanied by acute pancreatitis.

### 2.11. Treatment

After detailed discussion with the patient and obtaining his informed consent, we performed duodenum-preserving partial pancreatic head resection (DPPHR-P) combined with ligation from the root of the APD of the pancreas to treat the BD-IPMN. Intraoperative exploration showed that hyperemia and edema were localized in the uncinate duct of the pancreas. Severe adhesions of the uncinate process of the pancreas to the surrounding structures needed surgical removal. Meanwhile, the tumor’s “peduncle” – a branch of the duct of APD – was saddling just in front of the main pancreatic ducts and draining into APD. Thus, the region between the MD and APD was handled with care during the surgical excision of the tumor to protect the integrity of the main pancreatic ducts. Finally, a mass measuring 35*30*15 mm was successfully removed together with partial tissue of the pancreatic head and pancreas uncinate process, and the end of the APD near the MD was ligated simultaneously. Stomach, duodenum, common bile duct, and most of the pancreatic tissue and MD were preserved. Cross-section of the cystic tumor revealed small amounts of mucin secretion, and the openings of the branch pancreatic duct were identified macroscopically (Fig. 1C and D). Two drainage tubes were positioned at the dorsal and ventral regions of the pancreas at the end of the surgery.

### 2.12. Outcome and follow-up

The ventral drainage volume of the pancreas increased on the fourth day after surgery. The detection of high amylase levels in the drainage discharge (40713.5 U/L) led to the diagnosis of postoperative pancreatic fistula (POPF). The drainage volume was consistently >320 mL for 2 days, but the patient did not exhibit signs of peritoneal irritation. Given the low self-healing rate of POPF, the patient underwent endoscopic retrograde cholangiopancreatography plus endoscopic sphincterotomy plus endoscopic retrograde pancreatic, and pancreatic duct stenting. The dorsal drainage tube was removed after confirming a lack of fluid drainage on day 1 after surgery. The postoperative duration was uneventful and the patient was discharged 8 days after the surgery with the ventral drainage tube after the ventral drainage volume of the pancreas dropped to between 5 and 10 mL. Two weeks after discharge, the ventral drainage tube was removed (Fig. 1E and F).

**Figure 2.**
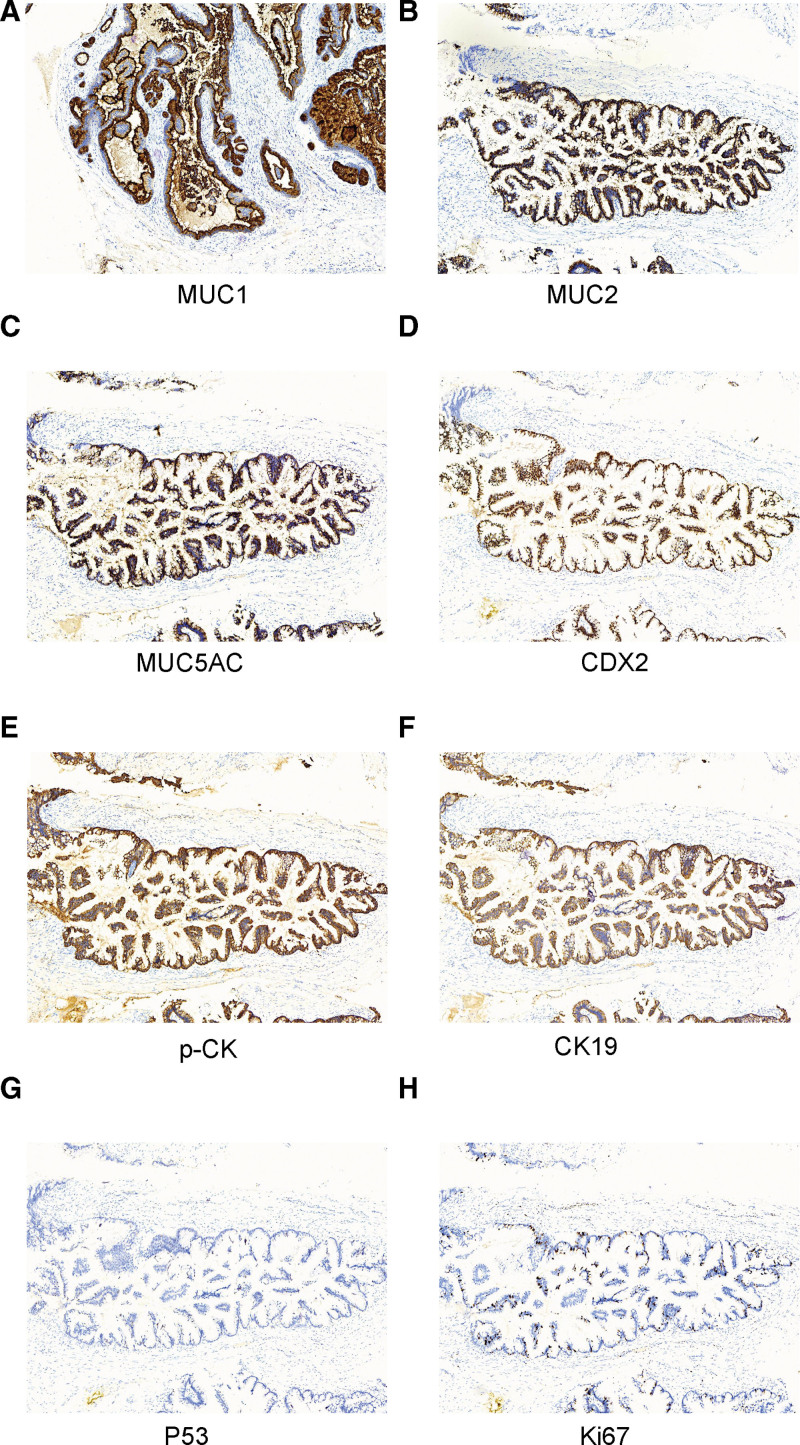
Immunohistochemical analysis. (A) the tumor cells were positive for MUC1; (B) MUC2; (C) MUC5AC; (D) CDX2; (E) p-CK, (F) CK19, (G) P53, and (H) Ki67 (10×). MUC = mucin core protein.

The presence of atypical epithelial cell clusters with papillary structure during the histological examination of the resected specimen was an indication that the BD-IPMN was characterized by high-grade intraepithelial neoplasia and local severe dysplasia of a pancreatobiliary subtype. Immunohistochemistry showed that the IPMN component was *MUC1* (+)/*MUC2* (+)/*MUC5AC* (+)/*CDX2* (+)/*p-CK* (+)/*CK19* (+)/*P53* (20%)/*Ki67* (15–30%) (Fig. 2).

## 3. Discussion

APD drains the anterior superior portion of the head of the pancreas by opening into the minor papilla. When there is excessive mucin secretion in the APD that drains through relatively fine-scaled APD and relatively small minor papilla in patients with IPMN, increased pressure in the APD leads to pancreatitis. The patient in the present case sought medical treatment due to the onset of acute pancreatitis. An imaging examination later confirmed that the change was caused by a 3.2 cm sized APD-IPMN cyst within the uncinate process of the pancreas, which meets the criteria for worrisome features. For pancreatic head tumors, especially for IPMC or IPMN with high risk for malignancy, pancreatoduodenectomy is the treatment of choice. However, for pancreatic head benign tumors, as our case presentation illustrates, a minimal DPPHR-P is likely to be the most favorable treatment approach. Although the “peduncle” of the tumor straddling the MD and the presence of severe adhesions significantly increased the surgical difficulty, DPPHR-P combined with ligation from the root of the APD of the pancreas successfully prevented the resection of a portion of the stomach, duodenum, and common bile duct, and protected the integrity of the main pancreatic ducts. This approach not only protected the physiological and anatomical integrity, but it also protected the endocrine and exocrine functions of the pancreas. Since the tumor grows longitudinally in the pancreatic duct, intraoperative rapid cryopathology is recommended to confirm that the cut margins are negative and ensure complete resection of the tumor. The scope of resection should be expanded or even total pancreatectomy should be performed if the following conditions exist: positive margins; high degree of heterogeneous growth in the margins; the tumor cannot be identified by freezing, and further testing is required.

POPF is the most frequent and harmful complication that occurs after partial pancreatic resection, with a reported incidence of 13.6 to 48%.^[14–16]^ Beger et al reported that limited pancreas head resection carries in terms of fistula B + C rate a low risk of postoperative complications.^[14,15]^ Lu et al reported that minimally invasive DPPHR is feasible and safe for benign or premalignant lesions of pancreatic head, but is associated with a high incidence of POPF (up to 48%).^[16]^ According to the ISGPS (version 2016) definition and grading, POPF can be categorized into 3 types: BL (biochemical leak, formerly grade A POPF: biochemical fistula), Grade B POPF and Grade C POPF. BL refers to increased amylase activity (> 3 × upper limit of normal) in the drainage fluid with no clinical implications, while Grade B indicates the presence of a pancreatic fistula associated with the incidence of clinically relevant conditions. The duration of pancreatic drainage can be extended to at least 3 weeks, and there may be a need to reposition the tubes placed during surgery through percutaneous or endoscopic imaging to reduce fluid pressure along the undrained fluid collection. Patients with Grade B POPF often require the administration of therapeutic agents to prevent infections that can cause organ failure or angiographic interventional procedures to prevent bleeding. Grade C POPF involves organ failure or even death, and possibly requires additional surgery.^[17]^ POPF is associated with longer hospital stays, increased risk of other invasive treatments and additional surgery, and even mortality. POPF is a main barrier to reducing postoperative morbidity and mortality related to pancreatic resection. The risk of POPF is higher for nonanatomic and limited resections.^[2]^ Similarly, our patient developed a pancreatic fistula on the fourth day after surgery. Pancreatic enzymes had not been activated by digestive fluid because of the integrity of the stomach, duodenum, and common bile duct, and the corrosive destruction of pancreatic leak fluid after DPPHR-P was weaker than those after pancreaticoduodenectomy. However, the high volume of drainage was a hindrance to spontaneous healing, and drainage management was necessary. Drainage management has been used to manage POPF, and includes image-guided (ultrasound or CT) percutaneous (external) and endoscopic (internal) drainage.^[18]^ External drainage requires daily care of the drainage tube and may easily lead to electrolyte loss, maldigestion, and infection. Therefore, we chose internal drainage through endoscopic pancreatic duct stenting. Stent placement significantly reduced the drainage volume of the abdominal drainage tube and helped to achieve spontaneous healing and avoid additional surgery. Pancreatic duct stenting facilitates pancreatic fistula recovery and can be performed during surgery to prevent POPF and iatrogenic injury of MD. However, it has limitations, like increased cost, induced pancreatitis, and exacerbated inflammation. Pancreatic duct stenting can also be performed after surgery for intensive treatment of POPF. In this way, the costs are reduced, and the treatment of POPF is individualized accurately. Postoperative pancreatic duct stenting also has its limitations, including amplified difficulty of the placement, need for additional surgery if it fails, and induced pancreatitis. Our case underscores that partial pancreatectomy combined with ligation from the root of the BD of the pancreas maximally protects the integrity of the main pancreatic ducts, and that endoscopic pancreatic duct stenting may be required if POPF appears after the operation.

IPMN can be classified into 4 subtypes (gastric, intestinal, pancreatobiliary, and oncocytic types) based on morphological features and immunohistochemistry for mucin core proteins (MUCs).^[2]^ MUC1 is a membrane-associated mucin that is known to contribute to epithelial cell-to-cell interactions and is predominantly expressed in the normal pancreas. *MUC2* is an important component of the mucous layer in the intestine and is not expressed in the normal pancreas. *MUC5AC* is frequently found in all IPMN subtypes and pancreatic intraepithelial neoplasia and is not detected in the normal pancreas. *MUC6* is expressed in both gastric and oncocytic tissue types. Studies have reported that the intestinal type is generally *MUC1* (−)/*MUC2* (+)/*MUC5AC* (+)/*MUC6* (−/weak+), while the gastric type is *MUC1* (−)/*MUC2* (−)/*MUC5AC* (+)/*MUC6* (+). The pancreatobiliary type is the least well-characterized and the least common, and the oncocytic type is defined by complex arborizing papillae with delicate cores, oncocytic cells, and intraepithelial luminal formation. Immunohistochemistry for MUCs shows *MUC1* (+)/*MUC2* (−)/*MUC5AC* (+)/*MUC6* (+) in pancreatobiliary and oncocytic types.^[2,19–21]^ Approximately 65% of the BD-IPMNs are of gastric type.^[22]^ However, if IPMN has a tendency towards malignant change, the histological subtypes of BD-IPMNs are usually tubular type.^[23]^ Our case was *MUC1* (+)/*MUC2* (+)/*MUC5AC* (+) BD-IPMN, suggesting he had the pancreatobiliary type. Meanwhile, *CDX2* (+)/*p-CK* (+)/*CK19* (+)/*P53* (20%)/*Ki67* (15–30%) further showed progress with atypical grade and invasive phenotype of the APD-IPMN. Pathohistological diagnosis revealed that APD-IPMN was high-grade intraepithelial neoplasia with local severe dysplasia. Therefore, APD-IPMN resection may be the best treatment option for selected patients. There is a need to systemically explore the clinicopathological and immunohistochemical characteristics of APD-IPMN in future studies.

## 4. Conclusion

APD-IPMN in the pancreas uncinate process has its own characteristics of localized pancreatitis, and MD-preserving DPPHR-P is the most effective method for its treatment as demonstrated in the present case report. This approach not only protects the exocrine and endocrine functions of the pancreas, but it also protects the physiological and anatomical integrity. The appearance of POPF after the DPPHR-P operation may require endoscopic pancreatic duct stenting. Additionally, the classification of APD-IPMN based on immunohistochemical analysis is crucial in selecting the appropriate diagnosis and treatment options.

## Acknowledgments

The authors are grateful to Dr Hao Zhang and Dr Hongqiang Hu for their valuable and constructive suggestions during the planning and development of this work.

## Author contributions

**Conceptualization:** Kun You, Cong Li.

**Data curation:** Tao Guo, Ya Liu, Zhu Yang, Jing Li, Kun You, Dejun Zhao, Sujuan Chen, Cong Li.

**Formal analysis:** Dejun Zhao.

**Investigation:** Kun You, Dejun Zhao, Pei Yang, Hao Zhang.**Methodology:** Zhu Yang, Dejun Zhao, Sujuan Chen, Pei Yang, Hao Zhang.**Project administration:** Tao Guo, Ya Liu, Zhu Yang, Jing Li, Hongqiang Hu, Hao Zhang.

**Resources:** Sujuan Chen, Hao Zhang.

**Software:** Ya Liu, Zhu Yang, Sujuan Chen, Cong Li, Pei Yang.

**Writing – original draft:** Tao Guo, Ya Liu, Zhu Yang.

**Writing – review & editing:** Hongqiang Hu, Hao Zhang.
